# Novel Autism Subtype-Dependent Genetic Variants Are Revealed by Quantitative Trait and Subphenotype Association Analyses of Published GWAS Data

**DOI:** 10.1371/journal.pone.0019067

**Published:** 2011-04-27

**Authors:** Valerie W. Hu, Anjene Addington, Alexander Hyman

**Affiliations:** 1 Department of Biochemistry and Molecular Biology, The George Washington University Medical Center, Washington, D.C., United States of America; 2 Child Psychiatry Branch, National Institute of Mental Health, National Institutes of Health, Bethesda, Maryland, United States of America; George Mason University, United States of America

## Abstract

The heterogeneity of symptoms associated with autism spectrum disorders (ASDs) has presented a significant challenge to genetic analyses. Even when associations with genetic variants have been identified, it has been difficult to associate them with a specific trait or characteristic of autism. Here, we report that quantitative trait analyses of ASD symptoms combined with case-control association analyses using distinct ASD subphenotypes identified on the basis of symptomatic profiles result in the identification of highly significant associations with 18 novel single nucleotide polymorphisms (SNPs). The symptom categories included deficits in language usage, non-verbal communication, social development, and play skills, as well as insistence on sameness or ritualistic behaviors. Ten of the trait-associated SNPs, or quantitative trait loci (QTL), were associated with more than one subtype, providing partial replication of the identified QTL. Notably, none of the novel SNPs is located within an exonic region, suggesting that these hereditary components of ASDs are more likely related to gene regulatory processes (or gene expression) than to structural or functional changes in gene products. Seven of the QTL reside within intergenic chromosomal regions associated with rare copy number variants that have been previously reported in autistic samples. Pathway analyses of the genes associated with the QTL identified in this study implicate neurological functions and disorders associated with autism pathophysiology. This study underscores the advantage of incorporating both quantitative traits as well as subphenotypes into large-scale genome-wide analyses of complex disorders.

## Introduction

Autism spectrum disorders (ASDs) represent a group of neurodevelopmental disorders that are characterized by impaired reciprocal social interactions, delayed or aberrant communication, and stereotyped, repetitive behaviors, often with restricted interests [1], [2]. With a concordance rate as high as 90% based on twin studies [3], ASDs are among the most heritable of neuropsychiatric conditions. Although specific genes have been found to be causal for several syndromes that are sometimes associated with autistic symptoms (for example, Fragile X [4], Retts [5], [6], and tuberous sclerosis [7], [8], there are no unequivocal genetic markers for non-syndromic idiopathic autism. Thus, a considerable amount of effort has been devoted to identifying genetic mutations or variants that associate with these perplexing and often devastating, life-long disorders. Because of the relatively high prevalence of ASDs in the general population (∼1∶110), genome-wide association (GWA) analyses have been used recently to search for common variants that may associate with increased susceptibility to this set of disorders [9]–[12]. However, despite case-control studies that have now exceeded many thousands of subjects and more than 500,000 SNPs, only a few significantly associated SNPs have been identified. In addition, replication of these SNPs in independent studies has not been successful. The inability to replicate findings from GWA analyses may be in part due to the genetic heterogeneity of the ASD population, thus giving rise to increased “noise” in the data. This genetic heterogeneity is likely responsible for the well-noted phenotypic and symptomatic heterogeneity among individuals with autism.

In a recent paper, we demonstrated that the ASD population can be divided into at least 4 phenotypic subgroups on the basis of cluster analyses of 123 severity scores taken from each individual's diagnostic assessment using the Autism Diagnostic Interview-Revised [13]. The resulting subgroups included one with severe language impairment, another with mild severity across all items, a third of intermediate severity, and a fourth of moderate severity with a higher frequency of savant skills. We further demonstrated by gene expression profiling of lymphoblastoid cell lines from 3 of these subgroups (excluding the intermediate) and nonautistic controls that cells from each of these subgroups exhibited differentially expressed genes relative to those of the controls, but also were distinguishable from each other in terms of unique, subtype-specific differentially expressed genes [14]. These studies thus support the concept that different subgroups of autistic individuals may exhibit subtype-dependent biological differences due to genetic variation. We therefore hypothesized that genetic association analyses of such ASD subtypes with SNPs that are identified and filtered according to their association with quantitative traits relevant to ASDs should reveal more significant SNPs with increased statistical power. Here, we report the identification of 18 novel and highly significant SNPs that are associated with at least one of 4 different subtypes of ASDs, based on the combination of quantitative trait association analyses and subtype-dependent genetic association analyses using trait-associated SNPs, with 10 of these SNPs replicated in a second ASD subtype representing an independent case cohort.

## Results

The overarching goal of these studies was to identify single nucleotide polymorphisms (SNPs) that are associated with both autistic traits and clinical subtypes of autism that are manifested by separate case cohorts. To accomplish this, we combined quantitative trait analysis and subphenotype association analyses using the wealth of genome-wide association (GWA) data published by Wang et al. in 2009 [9].

### Quantitative Trait Association Analyses

**Figure 1:**
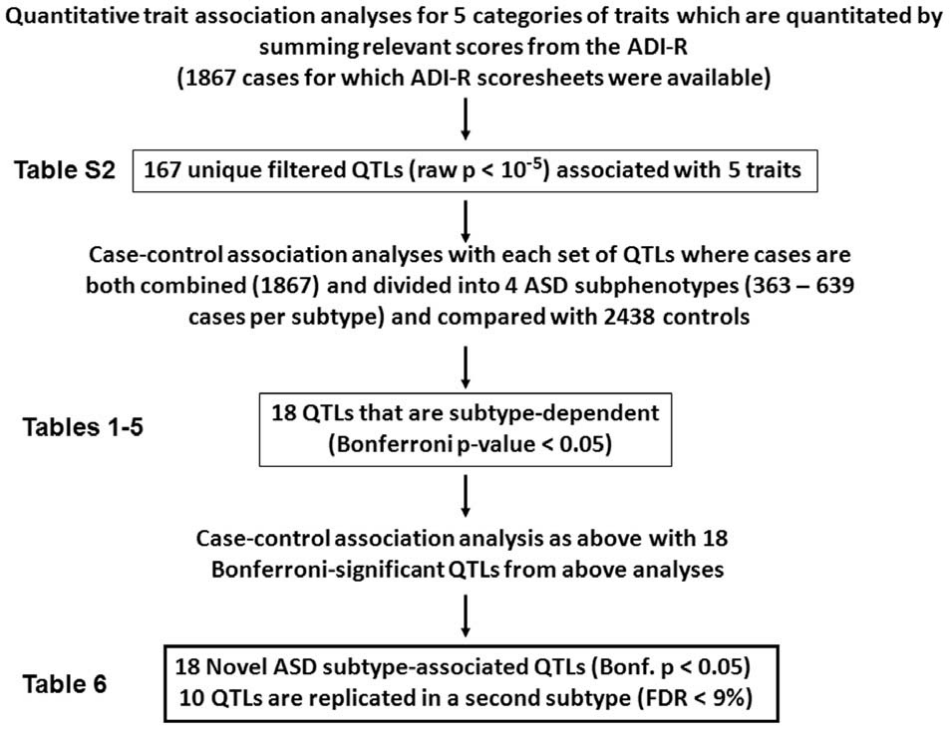
Diagram of study design illustrating sequential application of quantitative trait and subphenotype association analyses. The quantitative trait association analyses were performed with 1867 cases for which the ADI-R assessments were available, using the complete set of genetic polymorphisms to identify SNPs that may have functional relevance to traits associated with ASDs. This had the net effect of filtering the set of SNPs from 513,312 to 167 QTL. In the second phase, each set of trait-associated SNPs (QTL) were employed in case-control association analyses with 2438 controls and the combined cases (1867) as well as with 4 ASD subgroups, each representing an independent cohort of cases (363–639 cases per subtype). From these analyses, only 18 QTL with Bonferroni-adjusted p-values<0.05 in at least one subtype for each trait were combined for the final set of genetic association analyses using combined cases as well as ASD subtypes against the controls.

The flowchart in **Fig. 1** describes the experimental design and analyses that were used to derive the combined set of 18 novel and statistically significant SNPs that associate with 4 separate subtypes of ASDs. Raw item scores from the ADI-R score sheets of 2939 ASD cases were summed for spoken language skills, non-verbal communication, play skills, social development, and insistence on sameness/rituals, as described previously [13]. The specific items used to obtain the total score per “trait” category for each individual are shown in **Table S1** and the profiles of total scores for each category are shown for the 2939 individuals in **Fig. S1**. We then conducted quantitative trait association analyses using the distribution of scores in each of the categories to identify sets of SNPs that associate with symptomatic severity of each of the behaviors listed in Table S1. A nominal p-value≤10^−5^ was used in this “discovery phase” designed to screen candidate SNPs that are potentially of functional significance with respect to the ASD traits. These sets of symptom-associated SNPs (or quantitative trait loci, QTL) are shown in **Table S2**.

### ASD subtype-dependent genetic association analyses with trait-associated SNPs

Next, we performed cluster analyses as described by Hu and Steinberg [13] to divide the autistic cases into 4 non-overlapping phenotypic subgroups according to symptomatic severity profiles derived from 123 items on the ADI-R assessments. We postulated that this subtyping procedure, which reduces the behavioral/symptomatic heterogeneity among the cases within each subgroup, is likely to also restrict the genetic heterogeneity within each subgroup. The results of K-means cluster analyses (K = 4) of the ADIR data from the 2939 individuals (which included 1867 genotyped cases from the GWA study) is shown in **Fig. S2A**. An unsupervised principal components analysis (PCA) was used to demonstrate that the 4 subgroups of individuals with ASDs from the K-means cluster analyses results in effective separation of cases according to similarity of symptomatic profile as defined by the 123 item scores from the ADI-R (**Fig. S2B**). The resulting phenotypic subgroups were then used in genetic association analyses with each set of QTL derived from the five quantitative trait association analyses, where the cases were either divided into the 4 ASD subtypes or used as a combined case group against the 2438 nonautistic controls. These analyses produced 5 sets of QTL that were associated with specific subtypes of ASDs (**Tables 1** **,** **2** **,** **3** **,** **4** **,** **5**). It is interesting to note that SNPs associated with certain genes (such as HTR4, BACH2, and TINAG among Language QTL, and OR5B3, OR5B17, and ERBB4 among Nonverbal QTL) are replicated in more than one ASD subtype. Finally, significant SNPs with Bonferroni-adjusted p-values≤0.05 from each of the 4 separate subtype-dependent association analyses with QTL were combined into a single set containing 18 unique SNPs, and case-control association analyses were performed for which the cases were either combined into one group or divided according to subphenotype.

**Table 1:** Language QTL associated with ASD subtypes.

SNP	SNP position	Band	Location	Gene	UNADJ P	FDR_BH^a^	BONF^b^	Subtype
rs2277049	chr5:147883281	5q33.1	Intron	*HTR4*	0.0002	0.0015	0.0036	Language-impaired
rs757099	chr9:25727567	9p21.2	CNV*		0.0003	0.0015	0.0055	"
rs7785107	chr7:10247968	7p21.3	CNV		0.0003	0.0015	0.0059	"
rs7725785	chr5:147882896	5q33.1	Intron (boundary)	*HTR4*	0.0004	0.0015	0.0062	"
rs2287581	chr5:31341022	5p13.3	Intron (boundary)	*CDH6*	0.0007	0.0019	0.0111	"
rs1231339	chr9:25730863	9p21.2	CNV		0.0007	0.0019	0.0116	"
rs2180055	chr22:47722427	22q13.32	CNV		0.0015	0.0036	0.0252	"
rs758158	chr12:1895465	12p13.33	Intron	*CACNA2D4*	0.0032	0.0067	0.0538	"
rs17830215	chr8:1902159	8p23.3	Promoter	*KBTBD11*	0.0043	0.0082	0.0739	"
rs10183984	chr2:167181162	2q24.3	CNV		0.0060	0.0101	0.1013	"
rs11969265	chr6:90739765	6q15	Intron	*BACH2*	0.0080	0.0124	0.1359	"
rs9474831	chr6:54361040	6p12.1	Intron	*TINAG*	0.0099	0.0141	0.1688	"
rs17828521	chr6:54328202	6p12.1	Intron	*TINAG*	0.0116	0.0145	0.1977	"
rs10806416	chr6:90755541	6q15	Intron	*BACH2*	0.0120	0.0145	0.2036	"
rs6454792	chr6:90743794	6q15	Intron	*BACH2*	0.0349	0.0395	0.5927	"
rs12893752	chr14:25220350	14q12	CNV		0.0918	0.0975	1.0000	"
rs7785107	chr7:10247968	7p21.3	CNV		0.0016	0.0265	0.0265	Intermediate
rs12407665	chr1:14070302	1p36.21	CNV		0.0005	0.0093	0.0093	Mild
rs12893752	chr14:25220350	14q12	CNV		0.0068	0.0576	0.1151	"
rs6454792	chr6:90743794	6q15	Intron	*BACH2*	0.0137	0.0747	0.2330	"
rs1231339	chr9:25730863	9p21.2	CNV		0.0215	0.0747	0.3662	"
rs9474831	chr6:54361040	6p12.1	Intron	*TINAG*	0.0220	0.0747	0.3736	"
rs10183984	chr2:167181162	2q24.3	CNV		0.0356	0.0928	0.6059	"
rs17828521	chr6:54328202	6p12.1	Intron	*TINAG*	0.0382	0.0928	0.6495	"
rs757099	chr9:25727567	9p21.2	CNV		0.0441	0.0938	0.7503	"
rs7725785	chr5:147882896	5q33.1	Intron (boundary)	*HTR4*	0.0508	0.0960	0.8639	"

a p-value adjusted using Benjamini-Hochberg False Discovery Rate method;

b Bonferroni-adjusted p-value.

*CNV located in Chromosomal band as reported by Pinto et al. [15].

**Table 2:** Nonverbal communication QTL associated with ASD subtypes.

SNP	SNP Position	Band	Location	Gene	UNADJ	FDR_BH^a^	BONF^b^	Subtype
rs1231339	chr9:25730863	9p21.2	CNV*		0.0007	0.0177	0.0177	Language-impaired
rs519514	chr7:78294084	7q21.11	Intron	*MAGI2*	0.0031	0.0397	0.0793	"
rs11138895	chr9:82693023	9q21.31	-		0.0046	0.0401	0.1203	"
rs564127	chr7:79734155	7q21.11	CNV		0.0065	0.0423	0.1690	"
rs12466917	chr2:207427129	2q33.3	-		0.0159	0.0828	0.4141	"
rs11229411	chr11:57926918	11q12.1	Coding exon	*OR5B3*	0.0096	0.0898	0.2488	Intermediate
rs11229413	chr11:57927314	11q12.1	Coding exon	*OR5B3*	0.0102	0.0898	0.2646	"
rs12279895	chr11:57926572	11q12.1	Coding exon	*OR5B3*	0.0108	0.0898	0.2806	"
rs11229410	chr11:57926867	11q12.1	Coding exon	*OR5B3*	0.0138	0.0898	0.3590	"
rs1938670	chr11:57884219	11q12.1	Promoter	*OR5B17*	0.0176	0.0916	0.4579	"
rs1938651	chr11:57869527	11q12.1	CNV		0.0046	0.0511	0.1190	Moderate
rs1938672	chr11:57885629	11q12.1	Promoter	*OR5B17*	0.0060	0.0511	0.1560	"
rs11229410	chr11:57926867	11q12.1	Coding exon	*OR5B3*	0.0084	0.0511	0.2186	"
rs1938670	chr11:57884219	11q12.1	Promoter	*OR5B17*	0.0094	0.0511	0.2441	"
rs11229413	chr11:57927314	11q12.1	Coding exon	*OR5B3*	0.0112	0.0511	0.2919	"
rs11229411	chr11:57926918	11q12.1	Coding exon	*OR5B3*	0.0123	0.0511	0.3190	"
rs12279895	chr11:57926572	11q12.1	Coding exon	*OR5B3*	0.0138	0.0511	0.3580	"
rs9941626	chr2:212059912	2q34	Intron	*ERBB4*	0.0170	0.0554	0.4431	"
rs13021324	chr2:212058490	2q34	Intron	*ERBB4*	0.0288	0.0831	0.7483	"
rs7930778	chr11:57792932	11q12.1	Promoter	*OR10W1*	0.0341	0.0887	0.8866	"
rs730168	chr16:73707776	16q23.1	Intron	*LDHD*	0.0002	0.0036	0.0040	Mild
rs11671930	chr19:8023308	19p13.2	Promoter	*CCL25*	0.0003	0.0036	0.0073	"
rs13205238	chr6:413597	6p25.3	CNV		0.0033	0.0287	0.0860	"
rs12962411	chr18:27645521	18q12.1	CNV		0.0107	0.0522	0.2784	"
rs393076	chr3:162584791	3q26.1	CNV		0.0143	0.0522	0.3715	"
rs11229411	chr11:57926918	11q12.1	Coding exon	*OR5B3*	0.0146	0.0522	0.3794	"
rs11229413	chr11:57927314	11q12.1	Coding exon	*OR5B3*	0.0156	0.0522	0.4050	"
rs11229410	chr11:57926867	11q12.1	Coding exon	*OR5B3*	0.0191	0.0522	0.4961	"
rs4804202	chr19:12579619	19p13.2	Promoter	*FLJ90396*	0.0203	0.0522	0.5282	"
rs1231339	chr9:25730863	9p21.2	CNV		0.0215	0.0522	0.5600	"
rs1938670	chr11:57884219	11q12.1	Promoter	*OR5B17*	0.0221	0.0522	0.5737	"
rs12279895	chr11:57926572	11q12.1	Coding exon	*OR5B3*	0.0252	0.0530	0.6554	"
rs11138895	chr9:82693023	9q21.31	-		0.0265	0.0530	0.6887	"
rs1938651	chr11:57869527	11q12.1	CNV		0.0288	0.0535	0.7490	"
rs9941626	chr2:212059912	2q34	Intron	*ERBB4*	0.0337	0.0555	0.8772	"
rs1938672	chr11:57885629	11q12.1	Promoter	*OR5B17*	0.0342	0.0555	0.8886	"
rs4527692	chr6:21268426	6p22.3	Intron	*CDKAL1*	0.0376	0.0574	0.9764	"
rs665036	chr1:61053139	1p31.3	CNV		0.0412	0.0595	1.0000	"
rs7930778	chr11:57792932	11q12.1	Promoter	*OR10W1*	0.0464	0.0634	1.0000	"
rs13021324	chr2:212058490	2q34	Intron	*ERBB4*	0.0569	0.0706	1.0000	"
rs3133855	chr11:120090061	11q23.3	Intron	*GRIK4*	0.0570	0.0706	1.0000	"

a p-value adjusted using Benjamini-Hochberg False Discovery Rate method;

b Bonferroni-adjusted p-value.

*CNV located in Chromosomal band as reported by Pinto et al. [15].

**Table 3:** Play skills QTL associated with ASD subtypes.

SNP	SNP Position	Band	Location	Gene	UNADJ P	FDR_BH^a^	BONF^b^	Subtype
rs3754741	chr2:173761465	2q31.1	Intron	*ZAK*	0.0016	0.0641	0.1025	Language-impaired
rs9569991	chr13:33121745	13q13.2	-		0.0025	0.0641	0.1614	"
rs4798405	chr18:5732206	18p11.31	CNV*		0.0030	0.0641	0.1923	"
rs8181738	chr12:23095146	12p12.1	-		0.0045	0.0726	0.2906	"
rs1554547	chr12:97892279	12q23.1	Intron	*ANKS1B*	0.0068	0.0865	0.4324	"
rs1481513	chr8:79171163	8q21.12	CNV		0.0084	0.0893	0.5356	"
rs730168	chr16:73707776	16q23.1	Intron	*LDHD*	0.0002	0.0060	0.0098	Mild
rs6482516	chr10:18879356	10p12.33	Intron	*NSUN6*	0.0002	0.0060	0.0123	"
rs11671930	chr19:8023308	19p13.2	Promoter	*CCL25*	0.0003	0.0060	0.0179	"
rs11082277	chr18:38304361	18q12.3	CNV		0.0016	0.0221	0.1053	"
rs6698676	chr1:84528583	1p31.1	Promoter	*SAMD13*	0.0017	0.0221	0.1105	"
rs1461710	chr18:38197191	18q12.3	CNV		0.0029	0.0265	0.1877	"
rs3745651	chr19:12553001	19p13.2	Coding exon	*ZNF490*	0.0030	0.0265	0.1920	"
rs13205238	chr6:413597	6p25.3	CNV		0.0033	0.0265	0.2117	"
rs4386512	chr3:177012791	3q26.31	Downstream	*NAALADL2*	0.0048	0.0310	0.3067	"
rs6791089	chr3:176998117	3q26.31	Intron	*NAALADL2*	0.0052	0.0310	0.3342	"
rs9536962	chr13:54576937	13q21.1	CNV		0.0057	0.0310	0.3638	"
rs2250595	chr12:45642394	12q13.11	-		0.0058	0.0310	0.3716	"
rs4894734	chr3:177013204	3q26.31	Downstream	*NAALADL2*	0.0075	0.0342	0.4794	"
rs1481513	chr8:79171163	8q21.12	CNV		0.0079	0.0342	0.5064	"
rs7337921	chr13:23381053	13q12.12	Intron	*FLJ46358*	0.0080	0.0342	0.5124	"
rs1863080	chr2:36379940	2p22.3	CNV		0.0094	0.0365	0.5996	"
rs7944323	chr11:78903676	11q14.1	CNV		0.0097	0.0365	0.6206	"
rs10987251	chr9:128142815	9q33.3	Intron (boundary)	*C9orf28*	0.0151	0.0508	0.9679	"
rs6974649	chr7:130463637	7q32.3	CNV		0.0156	0.0508	1.0000	"
rs4894733	chr3:177013074	3q26.31	Downstream	*NAALADL2*	0.0166	0.0508	1.0000	"
rs9508456	chr13:28914362	13q12.3	Intron	*KIAA0774*	0.0172	0.0508	1.0000	"
rs1796045	chr2:96986737	2q11.2	CNV		0.0175	0.0508	1.0000	"
rs11229410	chr11:57926867	11q12.1	Coding exon	*OR5B3*	0.0191	0.0529	1.0000	"
rs4745257	chr9:75582705	9q21.13	CNV		0.0202	0.0529	1.0000	"
rs12606567	chr18:48408860	18q21.2	Intron	*DCC*	0.0211	0.0529	1.0000	"
rs2044747	chr14:42393956	14q21.2	CNV		0.0215	0.0529	1.0000	"
rs4646421	chr15:72803245	15q24.1	Intron	*CYP1A1*	0.0251	0.0595	1.0000	"
rs1440423	chr5:34425616	5p13.2	CNV		0.0269	0.0600	1.0000	"
rs1888156	chr9:128142661	9q33.3	Coding exon	*C9orf28*	0.0272	0.0600	1.0000	"
rs9941626	chr2:212059912	2q34	Intron	*ERBB4*	0.0337	0.0720	1.0000	"
rs2078520	chr2:80246156	2p12	Intron	*CTNNA2*	0.0376	0.0776	1.0000	"
rs1796028	chr2:96982680	2q11.2	CNV		0.0417	0.0834	1.0000	"
rs1482930	chr15:79651018	15q25.2	CNV		0.0488	0.0946	1.0000	"
rs11627027	chr14:93440170	14q32.13	-		0.0529	0.0995	1.0000	"

a p-value adjusted using Benjamini-Hochberg False Discovery Rate method;

b Bonferroni-adjusted p-value.

*CNV located in Chromosomal band as reported by Pinto et al. [15].

**Table 4:** Insistence on sameness QTL associated with ASD subtypes.

SNP	SNP Position	Band	Location	Gene	UNADJ	FDR_BH^a^	BONF^b^	Subtype
rs1827924	chr2:228377979	2q36.3	Promoter	*CCL20*	0.0002	0.0053	0.0053	Moderate
rs17738966	chr14:54371969	14q22.2	Downstream	*GCH1*	0.0005	0.0071	0.0171	"
rs7950390	chr11:4587928	11p15.4	Promoter	*TRIM68*	0.0007	0.0071	0.0212	"
rs3861787	chr11:4604346	11p15.4	CNV*		0.0012	0.0078	0.0385	"
rs317985	chr5:66773558	5q13.1	CNV		0.0012	0.0078	0.0392	"
rs3804967	chr3:7479914	3p26.1	Intron	*GRM7*	0.0022	0.0119	0.0712	"
rs3804968	chr3:7477700	3p26.1	Intron	*GRM7*	0.0033	0.0131	0.1069	"
rs13096022	chr3:7465129	3p26.1	Intron	*GRM7*	0.0034	0.0131	0.1102	"
rs6782718	chr3:7462776	3p26.1	Intron	*GRM7*	0.0037	0.0131	0.1182	"
rs9568011	chr13:47606812	13q14.2	-		0.0047	0.0152	0.1518	"
rs164187	chr1:160615261	1q23.3	Promoter	*C1orf111*	0.0070	0.0205	0.2251	"
rs10781238	chr9:76384432	9q21.13	Intron	*RORB*	0.0101	0.0240	0.3221	"
rs9634811	chr13:47362176	13q14.2	-		0.0103	0.0240	0.3303	"
rs2469183	chr15:84992924	15q25.3	CNV		0.0105	0.0240	0.3363	"
rs317985	chr5:66773558	5q13.1	CNV		0.0023	0.0584	0.0727	Mild
rs1827924	chr2:228377979	2q36.3	Promoter	*CCL20*	0.0037	0.0584	0.1168	"
rs2574852	chr17:73003467	17q25.3	Intron	*SEPT9*	0.0057	0.0609	0.1826	"

a p-value adjusted using Benjamini-Hochberg False Discovery Rate method;

b Bonferroni-adjusted p-value.

*CNV located in Chromosomal band as reported by Pinto et al. [15].

**Table 5:** Social development QTL associated with ASD subtypes.

SNP	SNP Position	Band	Location	Gene	UNADJ P	FDR_BH^a^	BONF^b^	Subtype
rs12266938	chr10:3852940	10p15.1	CNV*		0.0001	0.0023	0.0023	Mild
rs10519124	chr2:67819501	2p14	-		0.0002	0.0040	0.0081	"
rs2297172	chr9:71563166	9q21.11	Intron	*PTAR1*	0.0006	0.0082	0.0245	"
rs2255313	chr12:102773924	12q23.3	CNV		0.0014	0.0150	0.0613	"
rs6698676	chr1:84528583	1p31.1	Promoter	*SAMD13*	0.0017	0.0150	0.0760	"
rs2519866	chr17:27859883	17q11.2	Intron	*MYO1D*	0.0020	0.0150	0.0900	"
rs10305860	chr4:148625337	4q31.23	Intron	*EDNRA*	0.0028	0.0176	0.1229	"
rs13205238	chr6:413597	6p25.3	CNV		0.0033	0.0182	0.1456	"
rs4873815	chr8:144796206	8q24.3	Promoter	*ZNF623*	0.0042	0.0182	0.1836	"
rs4832481	chr2:16986218	2p24.3	CNV		0.0045	0.0182	0.1959	"
rs30746	chr5:135366157	5q31.1	CNV		0.0046	0.0182	0.2006	"
rs10997162	chr10:67906707	10q21.3	Intron	*CTNNA3*	0.0054	0.0196	0.2357	"
rs3809282	chr12:110192180	12q24.11	Intron	*CUTL2*	0.0061	0.0205	0.2664	"
rs12115722	chr9:133418328	9q34.13	CNV		0.0069	0.0218	0.3049	"
rs10874468	chr2:96959718	2q11.2	CNV		0.0099	0.0277	0.4361	"
rs4809918	chr20:50734607	20q13.2	-		0.0106	0.0277	0.4642	"
rs12962411	chr18:27645521	18q12.1	CNV		0.0107	0.0277	0.4712	"
rs4959923	chr6:412773	6p25.3	CNV		0.0129	0.0316	0.5679	"
rs721087	chr2:4942340	2p25.2	CNV		0.0166	0.0385	0.7312	"
rs9479482	chr6:150399705	6q25.1	-		0.0180	0.0397	0.7937	"
rs10788819	chr1:150161717	1q21.3	-		0.0198	0.0414	0.8690	"
rs4646421	chr15:72803245	15q24.1	Intron	*CYP1A1*	0.0251	0.0502	1.0000	"
rs11138895	chr9:82693023	9q21.31	CNV		0.0265	0.0507	1.0000	"
rs11138885	chr9:82678684	9q21.31	-		0.0300	0.0550	1.0000	"
rs4905110	chr14:93432083	14q32.13	-		0.0329	0.0570	1.0000	"
rs2627468	chr8:3812607	8p23.2	Intron	*CSMD1*	0.0349	0.0570	1.0000	"
rs17192980	chr5:7030768	5p15.31	CNV		0.0350	0.0570	1.0000	"
rs10886048	chr10:118928872	10q25.3	CNV		0.0426	0.0660	1.0000	"
rs4811895	chr20:55639715	20q13.31	-		0.0435	0.0660	1.0000	"
rs13384439	chr2:187500106	2q32.1	CNV		0.0462	0.0678	1.0000	"
rs4416176	chr2:26536150	2p23.3	Intron	*OTOF*	0.0507	0.0708	1.0000	"
rs11627027	chr14:93440170	14q32.13	-		0.0529	0.0708	1.0000	"
rs12183587	chr6:150396301	6q25.1	-		0.0531	0.0708	1.0000	"
rs2151206	chr9:14844072	9p22.3	Intron	*FREM1*	0.0562	0.0727	1.0000	"
rs6022029	chr20:50708572	20q13.2	CNV		0.0606	0.0762	1.0000	"
rs1996893	chr9:14880268	9p22.3	Intron	*FREM1*	0.0726	0.0887	1.0000	"

a p-value adjusted using Benjamini-Hochberg False Discovery Rate method;

b Bonferroni-adjusted p-value.

*CNV located in Chromosomal band as reported by Pinto et al. [15].

### Partial replication of SNPs between subtypes of ASDs

**Figure 2:**
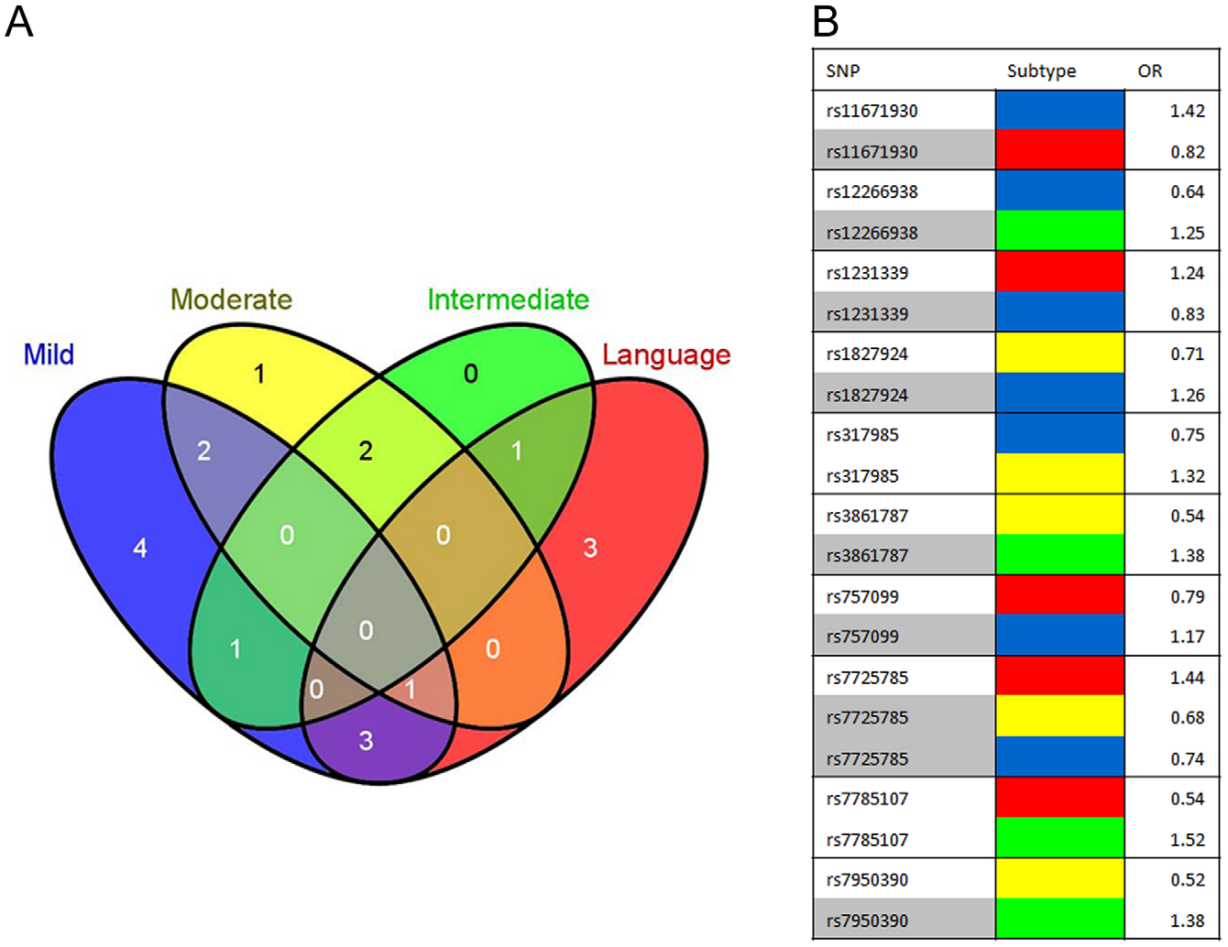
SNPs shared between subtypes exhibit different odds ratios. A) Venn diagram showing unique and shared SNPs across ASD subtypes. B) Table listing the shared SNPs and odds ratios in different ASD subtypes. Shading indicates SNPs with p-values that are <0.09 according to FDR_BH correction methods for multiple testing, while the unshaded SNPs have Bonferroni-adjusted p-values<0.05. For both (A) and (B), the subtypes are color-coded as follows: Red – Language-impaired; Green – Intermediate; Yellow – Moderate; Blue – Mild.

**Table 6** shows the SNPs associated with each subtype of ASDs that resulted from the final association analyses using the combined highly significant QTL and 4 non-overlapping subgroups of ASD cases. Eighteen of the SNPs have p-values<0.05 in at least one subtype even after using the stringent Bonferroni correction for multiple comparisons. Note that 10 of the SNPs, including rs317985, rs7785107, rs11671930, rs7950390, rs12266938, rs3861787, rs7725785, rs1827924, rs1231339, and rs757099, are associated with more than one subtype. Two of the replicated SNPs (rs317985 and rs7785107) are significant in two subtypes after Bonferroni adjustment (p<0.05), while the remaining 8 exhibit lower levels of significance (nominal p-values from 0.0037–0.051 or FDR_BH adjusted p-values of 0.0087–0.088) in the second (or third) subtype. Association of these QTL with more than one subtype of ASD serves as a replication for these 10 SNPs. While the replication of SNPs in different subtypes may be interpreted as evidence for those SNPs being highly penetrant for ASDs, the subtype-dependent differences in minor allele frequency (MAF) and odds ratios (OR) associated with the shared SNPs suggest that the subtypes are genetically heterogeneous. **Figure 2** summarizes the extent of SNP overlap among the 4 ASD subtypes (case cohorts) and clearly demonstrates that the odds ratios are distinctly different for different subtypes that share the same SNP. All of the QTL associated with specific genes are present in non-exonic (promoter, intronic, or intergenic) regions. Interestingly, all but one of the SNPs residing within intergenic regions can be associated by band position to rare copy number variants (CNV) that have been recently identified for ASDs [15], thus providing further support for the probable relevance of these SNPs to ASDs. These CNVs are noted in Table 6.

**Table 6:** Highly significant SNPs associated with ASD subtypes.

CHR	SNP	BP	A1^a^	F_A^b^	F_U^c^	A2^d^	CHISQ^e^	OR^f^	Band	Location	Gene	UNADJ	FDR_BH^g^	BONF^h^	Subtype (#cases)
5	rs2277049	147883281	*A*	0.115	0.081	*C*	13.71	1.48	5q33.1	Intron	*HTR4*	0.0002	0.0017	0.0041	Language (639)
9	rs757099	25727567	*C*	0.391	0.447	*T*	12.94	0.79	9p21.2	CNV*		0.0003	0.0017	0.0061	"
7	rs7785107	10247968	*T*	0.031	0.056	*G*	12.79	0.54	7p21.3	CNV		0.0003	0.0017	0.0066	"
5	rs7725785	147882896	*A*	0.114	0.082	*C*	12.71	1.44	5q33.1	Intron (boundary)	*HTR4*	0.0004	0.0017	0.0069	"
5	rs2287581	31341022	*A*	0.078	0.053	*G*	11.62	1.51	5p13.3	Intron (boundary)	*CDH6*	0.0007	0.0022	0.0124	"
9	rs1231339	25730863	*A*	0.534	0.480	*C*	11.54	1.24	9p21.2	CNV		0.0007	0.0022	0.0129	"
22	rs2180055	47722427	*T*	0.146	0.113	*C*	10.1	1.34	22q13.32	CNV		0.0015	0.0040	0.0281	"
19	rs11671930	8023308	*C*	0.135	0.160	*T*	4.809	0.82	19p13.2	Promoter	*CCL25*	0.0283	0.0598	0.5379	"
7	rs7785107	10247968	*T*	0.083	0.056	*G*	10.01	1.52	7p21.3	CNV		0.0016	0.0296	0.0296	Intermed (478)
11	rs7950390	4587928	*G*	0.107	0.080	*T*	7.51	1.38	11p15.4	Promoter	*TRIM68*	0.0061	0.0370	0.1166	"
10	rs12266938	3852940	*C*	0.236	0.198	*T*	7.14	1.25	10p15.1	CNV		0.0075	0.0370	0.1432	"
11	rs3861787	4604346	*T*	0.102	0.076	*G*	7.081	1.38	11p15.4	CNV		0.0078	0.0370	0.1480	"
2	rs1827924	228377979	*G*	0.256	0.326	*A*	14.2	0.71	2q36.3	Promoter	*CCL20*	0.0002	0.0031	0.0031	Moderate (363)
14	rs17738966	54371969	*A*	0.131	0.090	*G*	11.99	1.52	14q22.2	Downstream	*GCH1*	0.0005	0.0042	0.0102	"
11	rs7950390	4587928	*G*	0.044	0.080	*T*	11.59	0.53	11p15.4	Promoter	*TRIM68*	0.0007	0.0042	0.0126	"
11	rs3861787	4604346	*T*	0.043	0.076	*G*	10.49	0.54	11p15.4	CNV		0.0012	0.0047	0.0229	"
5	rs317985	66773558	*A*	0.321	0.264	*G*	10.45	1.32	5q13.1	CNV		0.0012	0.0047	0.0233	"
5	rs7725785	147882896	*A*	0.058	0.082	*C*	5.168	0.69	5q33.1	Intron (boundary)	*HTR4*	0.0230	0.0729	0.4372	"
10	rs12266938	3852940	*C*	0.137	0.198	*T*	16.33	0.64	10p15.1	CNV		0.0001	0.0009	0.0010	Mild (387)
16	rs730168	73707776	*A*	0.173	0.234	*G*	14.34	0.68	16q23.1	Intron	*LDHD*	0.0002	0.0009	0.0029	"
2	rs10519124	67819501	*A*	0.187	0.137	*G*	13.99	1.46	2p14			0.0002	0.0009	0.0035	"
10	rs6482516	18879356	*A*	0.310	0.247	*G*	13.91	1.37	10p12.33	Intron	*NSUN6*	0.0002	0.0009	0.0037	"
19	rs11671930	8023308	*C*	0.212	0.160	*T*	13.21	1.42	19p13.2	Promoter	*CCL25*	0.0003	0.0011	0.0053	"
9	rs2297172	71563166	*C*	0.093	0.139	*T*	11.92	0.64	9q21.11	Intron	*PTAR1*	0.0006	0.0018	0.0106	"
5	rs317985	66773558	*A*	0.212	0.264	*G*	9.317	0.75	5q13.1	CNV		0.0023	0.0062	0.0431	"
2	rs1827924	228377979	*G*	0.380	0.326	*A*	8.45	1.26	2q36.3	Promoter	*CCL20*	0.0037	0.0087	0.0693	"
9	rs1231339	25730863	*A*	0.436	0.480	*C*	5.283	0.83	9p21.2	CNV		0.0215	0.0455	0.4092	"
9	rs757099	25727567	*C*	0.486	0.447	*T*	4.051	1.17	9p21.2	CNV		0.0441	0.0839	0.8386	"
5	rs7725785	147882896	*A*	0.062	0.082	*C*	3.814	0.74	5q33.1	Intron (boundary)	*HTR4*	0.0508	0.0878	0.9655	"

a Minor allele;

b Minor allele frequency-autistic cases;

c MAF-unaffected controls;

d Major allele;

e ChiSquare value;

f Odds ratio with respect to major allele;

g p-value adjusted using Benjamini-Hochberg False Discovery Rate method;

h Bonferroni-adjusted p-value.

*CNV located in Chromosomal band as reported by Pinto et al. [15].

### Effect of subtyping of ASDs on association of previously identified SNPs within Chr5p14.1

Because none of the SNPs identified in this re-analysis overlapped with those of the previously published genome-wide association study [9], we examined the association of the 6 SNPs that were reported in the published study with our subphenotypes. **Table 7** shows that only the “Moderate” ASD subtype (363 cases) is associated with two of the SNPs, with Bonferroni-adjusted p-values of 0.035 and 0.053. Interestingly, these 2 SNPs have the least significant combined p-values in the published study. The remaining 4 SNPs were suggestively significant with FDR_BH-adjusted p-values of 0.074 in this subtype. The combined cases (1867 individuals in all) as well as the other 3 ASD subtypes show no association with any of the 6 SNPs even though there are more cases in each of these groups than in the Moderate group. This finding further illustrates the value of analyzing subphenotypes of ASDs in genome-wide association analyses in order to reduce the genetic heterogeneity of the population studied.

**Table 7:** Association of 6 SNPs from original GWA study [9] with ASD subtypes.

CHR	SNP	BP	A1^a^	MAF_A^b^	MAF_U^c^	A2^d^	CHISQ^e^	OR^f^	UNADJ P	FDR_BH^g^	BONF^h^	Subtype (# cases)
5	rs1896731	25934777	*C*	0.415	0.362	*T*	7.61	1.25	0.0058	0.0266	0.0348	Moderate (363)
5	rs10038113	25938099	*C*	0.471	0.420	*T*	6.85	1.23	0.0088	0.0266	0.0531	"
5	rs7704909	25934678	*C*	0.315	0.351	*T*	3.57	0.85	0.0589	0.0740	0.3533	"
5	rs4327572	26008578	*T*	0.314	0.349	*C*	3.38	0.85	0.0660	0.0740	0.3957	"
5	rs12518194	25987318	*G*	0.314	0.348	*A*	3.23	0.86	0.0725	0.0740	0.4350	"
5	rs4307059	26003460	*C*	0.312	0.345	*T*	3.19	0.86	0.0740	0.0740	0.4441	"
5	rs4307059	26003460	*C*	0.332	0.345	*T*	1.74	0.94	0.1870	0.3607	1.0000	Combined cases (1867)
5	rs12518194	25987318	*G*	0.334	0.348	*A*	1.68	0.94	0.1954	0.3607	1.0000	"
5	rs4327572	26008578	*T*	0.336	0.349	*C*	1.53	0.94	0.2155	0.3607	1.0000	"
5	rs7704909	25934678	*C*	0.339	0.351	*T*	1.38	0.95	0.2405	0.3607	1.0000	"
5	rs10038113	25938099	*C*	0.425	0.420	*T*	0.28	1.02	0.5978	0.7173	1.0000	"
5	rs1896731	25934777	*C*	0.365	0.362	*T*	0.10	1.02	0.7476	0.7476	1.0000	"
5	rs1896731	25934777	*C*	0.340	0.362	*T*	2.00	0.91	0.1576	0.4388	0.9456	Language-impaired (639)
5	rs10038113	25938099	*C*	0.402	0.420	*T*	1.28	0.93	0.2579	0.4388	1.0000	"
5	rs4307059	26003460	*C*	0.332	0.345	*T*	0.83	0.94	0.3629	0.4388	1.0000	"
5	rs4327572	26008578	*T*	0.336	0.349	*C*	0.77	0.94	0.3800	0.4388	1.0000	"
5	rs12518194	25987318	*G*	0.335	0.348	*A*	0.71	0.95	0.3991	0.4388	1.0000	"
5	rs7704909	25934678	*C*	0.340	0.351	*T*	0.60	0.95	0.4388	0.4388	1.0000	"
5	rs7704909	25934678	*C*	0.358	0.351	*T*	0.15	1.03	0.6983	0.9830	1.0000	Intermediate (478)
5	rs4327572	26008578	*T*	0.351	0.349	*C*	0.02	1.01	0.8895	0.9830	1.0000	"
5	rs12518194	25987318	*G*	0.346	0.348	*A*	0.01	0.99	0.9403	0.9830	1.0000	"
5	rs10038113	25938099	*C*	0.418	0.420	*T*	0.00	1.00	0.9469	0.9830	1.0000	"
5	rs1896731	25934777	*C*	0.361	0.362	*T*	0.00	1.00	0.9634	0.9830	1.0000	"
5	rs4307059	26003460	*C*	0.345	0.345	*T*	0.00	1.00	0.9830	0.9830	1.0000	"
5	rs7704909	25934678	*C*	0.337	0.351	*T*	0.57	0.94	0.4485	0.7486	1.0000	Mild (387)
5	rs4307059	26003460	*C*	0.334	0.345	*T*	0.37	0.95	0.5455	0.7486	1.0000	"
5	rs4327572	26008578	*T*	0.339	0.349	*C*	0.31	0.96	0.5759	0.7486	1.0000	"
5	rs12518194	25987318	*G*	0.337	0.348	*A*	0.31	0.96	0.5763	0.7486	1.0000	"
5	rs10038113	25938099	*C*	0.429	0.420	*T*	0.24	1.04	0.6239	0.7486	1.0000	"
5	rs1896731	25934777	*C*	0.364	0.362	*T*	0.02	1.01	0.8853	0.8853	1.0000	"

a Minor allele;

b Minor allele frequency-autistic cases;

c MAF-unaffected controls;

d Major allele;

e ChiSquare value;

f Odds ratio with respect to major allele;

g p-value adjusted using Benjamini-Hochberg False Discovery Rate method;

h Bonferroni-adjusted p-value*CNV located in Chromosomal band as reported by Pinto et al. [15].

### Pathway analyses of SNP-containing genes

**Figure 3:**
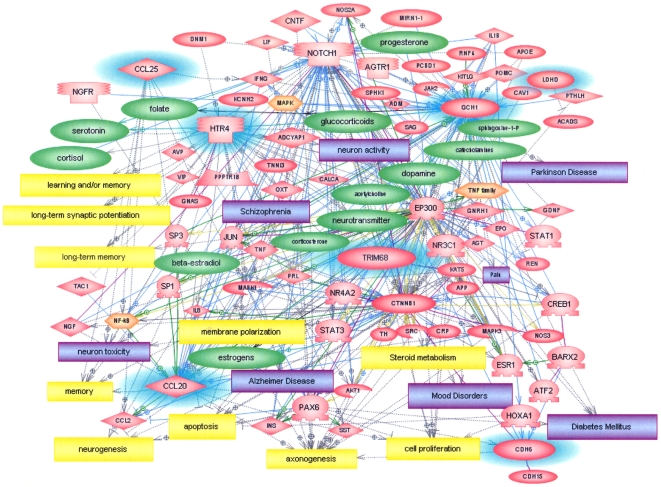
Gene interaction network of genes associated with the intronic SNPs identified in **Table 6**. Genes from Table 6 are shown with blue highlights while other genes in the network are shown in pink and small molecules are green. Processes are shown in yellow and disorders are shown in purple. The orange entities represent functional complexes.

To obtain a better understanding of how the novel SNPs identified in this study potentially relate to the biology of autism, pathway analysis was conducted to develop a better sense of the relationships among the SNP-associated genes and their impact on higher level functions and diseases. **Fig. 3** shows a gene network constructed using Pathway Studio 7 which includes seven of the 9 genes associated with SNPs found within gene promoters or introns. Of the 7 genes, HTR4 and GCH1 are “hubs” showing the highest “connectivity” with other components within the network. The specific relationships between these two genes and other network components are more clearly illustrated in **Figures S3 and S4**. It is noted that many of the cellular and higher level processes in this network, such as neurogenesis, axonogenesis, steroid metabolism, cell proliferation, long-term synaptic potentiation, learning and memory are relevant to identified deficits in ASDs.

## Discussion

We have previously shown that the autistic population can be divided into subgroups of affected individuals according to symptomatic profile through cluster analyses of severity scores from the ADI-R assessment for each individual with an ASD [13]. Three of the 4 resulting subgroups were shown to exhibit distinct, though partially overlapping, gene expression profiles, each relative to a group of nonautistic controls, implying that both unique and shared genes are associated with the respective phenotypes [14]. We therefore applied the same rationale and methods in subtyping individuals with ASDs for this re-analysis of previously published genome-wide association data [9]. We first employed quantitative trait association analyses to the >500,000 SNPs tested in order to prioritize SNPs that might correlate with a behavioral or symptomatic “trait” relevant to ASDs. These quantitative traits for each individual with an ASD were derived from the sums of severity scores from the ADI-R items that described severity of language impairment, deficits in nonverbal communication, impaired play skills, insistence on sameness/rituals, and delayed social development. The specific items used to establish each quantitative trait (listed in Table S1) were shown by Hu and Steinberg to exhibit differential severity among the several subtypes of ASDs [13]. In this “discovery” stage of the experiment, quantitative trait association analyses across the 5 selected traits produced a filtered set of 167 unique SNPs from the original 513,312 SNPs (**Table S2**). Subsequent association analyses of these QTL with both combined and subtyped individuals with ASDs revealed 18 novel SNPs which were found to be highly significant (Bonferroni-adjusted p-value<0.05) in at least one subtype of autism (**Table 6**). Interestingly, many of the language QTL from Table S2 are strongly associated with the severely language-impaired ASD subtype. Of the 18 novel SNPs, 10 SNPs were replicated, albeit at a more modest significance level, in at least one of the other subtypes. Two are replicated with Bonferroni-significant p-values while the other 8 are suggestively significant (FDR-BH adjusted p-value<0.09). While it may be argued that the standard statistical methods used here to correct for multiple testing are not stringent enough, there is still no clear consensus on the significance level that is appropriate for a candidate gene (or SNP) study such as this, where the candidate SNPs are determined by prior quantitative trait association analyses. More significantly, different minor allele frequencies and odds ratios are observed for the SNPs that are associated with more than one subtype (Table 6 and Fig. 2), thus reflecting the genetic heterogeneity among the ASD phenotypes, which is teased apart by the subtyping procedures employed here. It is noteworthy that no significant SNPs were identified when all 1867 individuals with ASDs were analyzed against 2438 nonautistic controls, thus underscoring the importance of phenotypic subtyping to unearthing SNP associations with ASDs.

By comparison, the original genome-wide study which was based on the combined analysis of 2503 cases and more than 7000 controls across 2 independent datasets in the “discovery” phase, identified 1 SNP that reached genome-wide significance and 5 additional SNPs of nominal significance in one intergenic region on chr5p14.1 that was located between 2 cadherin genes [9]. In this study, we replicated the association of 2 of the SNPs but only in the “Moderate” subtype of ASDs which consisted of only 363 cases (Table 7). Neither the other 3 subtypes (with 387–639 cases per subgroup) nor the combined cases group (consisting of 1867 cases) showed any association with these 6 SNPs. Furthermore, while none of the 6 SNPs identified in the published study correlated with expression level of either cadherin (CDH9 or CDH10) in the cortical brain of 93 genotyped human subjects, we note that 2 of the SNP-associated genes identified in the current study, HTR4 and CCL25, were found to be differentially expressed (FDR<5%) in lymphoblastoid cell lines from individuals with ASDs in a previous study by our laboratory [14]. This overlap of differentially expressed genes with those associated with at least some of the novel SNPs lends support to the potential functional relevance of these SNPs. Aside from the SNPs located within the promoter or intron regions of genes, seven of the other eight significant SNPs (Table 6) are located in intergenic regions that are linked by band position to rare copy number variants (CNVs) that have been recently associated with autism [15]. The finding that all 18 of the novel SNPs are in non-exonic (promoter and noncoding) regions further suggests that gene regulatory processes likely leading to differential gene expression, rather than to alterations of gene products, may contribute to the hereditary component of ASDs. The presence of the identified QTL in ASD-related CNVs provides additional support for the potential relevance of these novel SNPs to ASDs. The current study thus illustrates the advantage of combining both quantitative traits and defined ASD phenotypes in analyzing genome-wide genetic data from individuals with this complex disorder.

### Quantitative trait and phenotype incorporation into genetic analyses

There have been relatively few large-scale genetic studies on ASDs that incorporate quantitative traits or phenotypes into genome-wide analyses. The value of using phenotypic subsets of ASDs in genetic analyses was demonstrated in an early linkage study by Nurmi et al. who found that subsetting the subjects (and families) according to presence or absence of savant skills increased the HLOD score of the D15S511 marker within the GABRB3 gene on chr15q11–q13 from 0.8 in the entire study population to 2.6 for the savant-skills-positive subset of individuals [16]. Later, Alarcon et al. used quantitative trait analysis of 2 language scores on the ADI-R combined with ordered subsets analysis for subject stratification to reveal a strong language QTL on 7q35, and observed additional linkage peaks on several other chromosomes without stratification [17]. Chen et al. used a combination of 7 ADI-R scores describing nonverbal communication and the same approach to uncover QTL for nonverbal communication [18]. Similarly, Duvall et al. used quantitative trait analysis based upon scores from the Social Responsiveness Scale to discover QTL for social endophenotypes with strong linkage to chromosomes 11 and 17 across all samples and additional linkages to chromosomes 4, 8, and 10 in “male-only” families [19]. More recently, Nijmeijer et al. identified loci overlapping ASDs and attention-deficit/hyperactivity disorder (ADHD) in a genome-wide study of children with ADHD and autistic traits (but not classical or atypical autism) by using total and subscale scores from the Social Communication Questionnaire (an autism diagnostic instrument) as well as by including scores related to severity of ADHD as covariates [20]. Along a similar vein, Ronald et al. examined both social and non-social autistic-like traits in the general population, quantified on the basis of DSM-IV social and non-social scales, to tease out 22 and 20 SNPs that associated with these traits, respectively [21]. However, only one SNP survived testing in an independent sample and its p-value was still only nominally significant in an all male sample. Recently, quantitative trait analysis was applied in a genome-wide linkage study of two repetitive ASD behavior phenotypes, “insistence on sameness” (IS) and “repetitive and stereotyped behavior” (RSMA) [22]. In this study, Cannon et al. demonstrated IS and RSMA were linked to 2 separate loci, each exhibiting respective HLOD scores of 3.42 and 3.93, suggesting that more narrowly-defined phenotypes improve linkage signals. When social behavior, as reflected by severity scores on the Social Responsiveness Scale was used as a quantitative trait, Coon et al. not only replicated a susceptibility locus identified previously in the Duvall et al. study [19], but also identified additional linkage peaks with HLOD scores ranging from 2.23–3.64 [23]. Interestingly, St. Pourcain et al. recently reported that one of the SNPs identified at Chr5p14 by GWA analyses [9] also associates by quantitative trait analyses with the social communication phenotype [24]. They suggested that this SNP, rs4307059, may also associate with multiple subthreshold impairments across a variety of ASD-related functions.

In the present study, we observe that rs4307059 is suggestively associated only with the Moderate ASD subtype, with an unadjusted p-value of 0.074 (Table 7). Thus, the above-mentioned studies, in combination with ours, reinforce the concept that the incorporation of quantitative traits into genetic analyses greatly improves the ability to elicit significant genetic information on complex disorders such as ASDs. Furthermore, the genes associated with the QTL in Tables 1, 2, 3,4, 5 and Table S2 may provide clues to the biology underlying the various autistic traits. Our study, *which uniquely combines QTL with ASD subtype-dependent genetic association analyses*, provides an additional refinement to genome-wide studies that results not only in the identification of a greater number of significant SNPs, but also concomitant and internal validation of SNPs that are replicated between and among the ASD subtypes which represent independent cohorts of affected individuals. Since the number of individuals in each subgroup ranges from 363–639, our study also demonstrates the increased statistical power inherent in this approach. Clearly, there are many ways that individuals with ASDs can be divided into subgroups (such as by regression following normal development, co-morbid symptoms, IQ, etc.), and some of these methods may prove to be superior to the method described here and in our previous study [13]. However, the importance of using phenotypically distinct cohorts in large-scale genetic association analyses is illustrated here using a method that is capable of distinguishing meaningful phenotypes across the whole spectrum of ASDs (severe to mild), based on severity profiles across a broad range of behaviors and symptoms characteristic of ASDs.

### Biological relevance of SNP-containing genes

To examine the biological processes and pathways that might be impacted by the SNP-associated genes, we performed pathway analyses on the genes using Pathway Studio 7 software. Fig. 3 shows that 7 of the 9 SNP-containing genes are included in a gene network with many genes leading to “axonogenesis,” a function which has been reported to be enriched among genes overlapping copy number variants in autistic patients [25]. HTR4 and GCH1 are network “hubs” connecting with many other genes, cellular processes and disorders (see Figs. S3 and S4 for specific connections). As illustrated in Fig. S3, HTR4 [5-hydroxytryptamine (serotonin) receptor 4] regulates neurogenesis, long-term synaptic potentiation and, in turn, learning and memory, as well as the release of neurotransmitters (dopamine, acetylcholine), peptide hormones (AVP, OXT, PRL, VIP) and steroid compounds (cortisol, corticosterone). Thus, any alteration in the expression or function of this gene can be expected to have wide-ranging consequences on processes known to be affected by ASDs. It is notable that one of the SNPs associated with HTR4, rs7725785, is associated with three ASD subtypes (Table 6). However, the odds ratio is 1.44 for the severe language-impaired subtype while it is 0.68 and 0.74 for the moderate and mild subtypes, respectively. Interestingly, a reduction in HTR4 expression was observed only for the language-impaired subtype of ASDs and not for the moderate (savant) or mild subtypes in our earlier gene expression study [14], suggesting that this SNP, which is located at an intron boundary within HTR4, may play a role in level of expression. Genetic variants in HTR4 have also been associated with schizophrenia [26], bipolar disorder [27] and attention deficit/hyperactivity disorder [28], further suggesting some functional overlap among these neurological disorders. More recently, a *de novo* translocation on chromosome 5 close to HTR4 has been identified in an autistic boy [29], lending additional support for the relevance of this gene in ASDs.

The other hub gene, GCH1 [GTP cyclohydrolase I], is the rate-limiting enzyme in the *de novo* biosynthesis of tetrahydrobiopterin which is, in turn, required for the biosynthesis of folate, serotonin, dopamine, and catecholamines (Fig. S4), all of which are important for neural development and functions. It is interesting to note that elevated expression of GCH1 has been implicated in mood disorders [30], while genetic polymorphisms or mutations in GCH1 have been associated with pain sensitivity [31]–[33], and dystonia [34], which are often associated with ASDs. Although these genes are not likely to be causal for ASDs, genetic polymorphisms in them may be associated with some of the comorbid symptoms or pathobiology of ASDs.

Together, these analyses provide support for the possible biological relevance of these QTL to ASDs and identify additional candidate genes, such as HTR4 and GCH1, for functional testing. Importantly, this study also reveals genetic biomarkers which not only may be used for diagnostic screening of ASDs, but also offer the additional advantage of being associated with ASD subtypes that may be linked to specific and targeted therapies through pharmacogenomics studies. Finally, the association of different SNPs with the 4 subtypes of ASDs described here reinforces the idea that there are multiple genetic etiologies giving rise to the autistic spectrum, while the shared SNPs between different subtypes may reveal common genetic mechanisms responsible for core symptoms.

### Summary

This study is the first to demonstrate the value of using a combination of quantitative trait analysis and subphenotyping of individuals with ASDs to identify genetic variants (SNPs) that associate with specific behavioral phenotypes of ASDs. It is noted that no Bonferroni-significant SNPs are detected when all 1867 autistic cases are combined into a single group and compared against 2438 non-autistic controls. Thus, even though the number of cases is lower in each of the subgroups, there is more power to detect statistically significant SNPs associated with the more homogeneous subgroups of individuals with ASDs than with the combined group of cases. Subtyping also creates independent case cohorts which are shown to replicate 10 of the novel SNPs identified in this study, thus providing a form of internal validation. Differences in minor allele frequencies of these 10 SNPs in the different cohorts further demonstrate the genetic heterogeneity among the subtypes. Together, these findings not only reveal novel subtype-dependent candidate genes for ASDs, but also identify potential genetic markers for diagnostic screening.

## Methods

### GWA data used for this study

Genome-wide association data from the study by Wang et al. [9] were downloaded from the Autism Speaks website at ftp://ftp.autismspeaks.org/Data/CHOP_PLINK/AGRERELEASE.ped. For this study, we used the file named CHOP.clean100121 where the data were “cleaned” by Jennifer K. Lowe in the laboratory of Daniel H. Geschwind, M.D., Ph.D. at UCLA. The cleaning procedure involved extensive sample and pedigree validation, exclusion of SNPs a) missing >10% data, b) with HWE p<0.001, c) with MAF<0.01, and d) with >10 Mendelian errors. The final dataset included 4327 genotyped individuals and 513,312 SNPs on the Illumina HumanHap550 Bead Chip.

### ADI-R data

ADI-R assessments for 2939 individuals were obtained from Autism Speaks through Dr. Vlad Kustanovich of the Autism Genetics Resource Exchange. Of these, 1867 individuals were among the cases genotyped by Wang et al. [9]. Scores of 123 items on the ADI-R score sheets of each individual were analyzed as described by Hu and Steinberg [13] to identify ASD subtypes (that is, phenotypic subgroups) which are represented in **Fig. S2**.

### Determination of quantitative traits

Raw item scores from the Autism Diagnostic Interview-Revised (ADI-R) score sheets of 2939 ASD cases were summed for spoken language skills, non-verbal communication, play skills, social development, and insistence on sameness/ritualistic behaviors. The specific items used to obtain the total score per category for each individual, shown in **Table S1**, were noted in an earlier study [13] to exhibit average differences in severity among several subtypes of ASDs, described below. The sums of item scores within each of the 5 categories were used as quantitative traits for genetic association analyses using the genotype data reported by Wang et al. [9]. Profiles of the traits across the 2939 individuals are shown in **Fig. S1**.

### Subtyping of individuals with ASDs

Two thousand nine hundred and thirty nine (2939) individuals with ASDs were divided into phenotypic subgroups using clustering tools within the MeV software package [35], as previously described by Hu and Steinberg [13]. Briefly, subtyping of individuals with ASDs involved K-means cluster analysis (with K = 4) of scores from 123 items from the ADI-R score sheets of each individual which were adjusted as described to fall into a severity range of 0 (normal) to 3 (highest severity). A figure of merit analysis (not shown) indicated that the individuals with ASDs were optimally represented by 4 subgroups. A principal components analysis (PCA) was then used to verify that the 4 subgroups of individuals identified by the K-means cluster analyses were distinguishable by this unsupervised test. **Fig. S2** shows the symptomatic profiles of the 4 ASD subtypes as well as their separation into discernible clusters by PCA. The subtypes are named “Language-impaired,” “Intermediate,” “Moderate,” and “Mild,” and contain 639, 478, 363, and 387 cases, respectively.

### Quantitative trait association analyses

Using the score totals for the 5 categories of autistic symptoms exhibited by each of 1867 cases as quantitative traits, we utilized PLINK [36], a program to analyze whole genome association data, to perform quantitative trait association analyses with the genotype data reported by Wang et al. [9]. From the results of each of the 5 analyses (**Table S2**) in this discovery phase, we selected SNPs with unadjusted p-values<10^−5^, which prioritized SNPs filtered by association with quantitative traits relevant to ASDs. These filtered sets of SNPs were then used in case-control association analyses, as described below.

### Case-control association tests

Using the 5 sets of QTL generated for each symptom category, association analyses using PLINK were performed with each set where the cases, in addition to being treated as a single group of 1867 cases vs. 2438 controls, were also divided into the 4 ASD subtypes that were determined by the ADI-R cluster analyses described above. It should be noted that each ASD subgroup represents an entirely separate cohort of cases. From each of the 5 sets of genetic association analyses with subtypes and QTL (**Tables 1** **,** **2** **,** **3** **,** **4** **,** **5**), we selected SNPs associated with each ASD subtype with a Bonferroni-adjusted p-value≤0.05 and combined them (a total of 18 unique SNPs) for a second case-control association analysis using the combined and subtyped ASD cases against controls. The results of this final analysis are shown in **Table 6**.

### Pathway analysis

Pathway Studio 7 software (Ariadne Genomics, Inc.) was used to generate relational gene networks using the SNP-containing genes listed in Table 6.

## Supporting Information

Figure S1 **Quantitative trait profiles generated by summing the severity scores for ADI-R items for each trait listed in Table S1.**
(TIF)

Figure S2 **Identification of ASD subtypes by cluster analyses.** A) Symptomatic profiles of the 4 ASD subtypes that resulted from K-means cluster analyses of 123 ADI-R severity scores per individual. In this figure, each row represents an individual and each column represents an item on the ADI-R. Black represents a score of 0 which is considered “normal,” while the intensity of red indicates severity scores ranging from 1–3. Gray represents unavailable data. The wide band of intensely red items in the language-impaired subgroup corresponds to spoken language. The 12 columns at the extreme right in each block represent items corresponding to “Savant skills,” which appear to be present at a slightly higher frequency in the group labeled “Moderate.” This group had been labeled “Savant” in our previous study [13]. Note that each cluster contains an independent cohort of cases. B) Principal components analysis (PCA) of the individuals based on the 123 ADI-R severity scores. Each subgroup of individuals identified in (A) is assigned a color, which identifies individuals from that subgroup in the PCA. Red: Language-impaired; Green: Intermediate; Yellow: Moderate; Blue: Mild. Each point on the PCA represents an individual with an ASD whose position is defined by his/her scores for the 123 ADI-R items.
(TIF)

Figure S3 **Network connections centered on HTR4 from** **Figure 3** **.**
(TIF)

Figure S4 **Network connections centered on GCH1 from** **Figure 3** **.**
(TIF)

Table S1 **List of behavioral categories and associated ADI-R items used for quantitative trait (QT) analyses.**
(DOC)

Table S2 **Quantitative trait loci associated with 5 ASD traits.** Language-impairment, deficits in nonverbal communication, impaired play skills, insistence on sameness and rituals, and delayed social development.
(XLS)
