# Supplementary figures and images for: Novel Autism Subtype-Dependent Genetic Variants Are Revealed by Quantitative Trait and Subphenotype Association Analyses of Published GWAS Data
Source: PLoS One. 2011 Apr 27;6(4):e19067. doi: 10.1371/journal.pone.0019067 (PMC3083416; doi:10.1371/journal.pone.0019067)

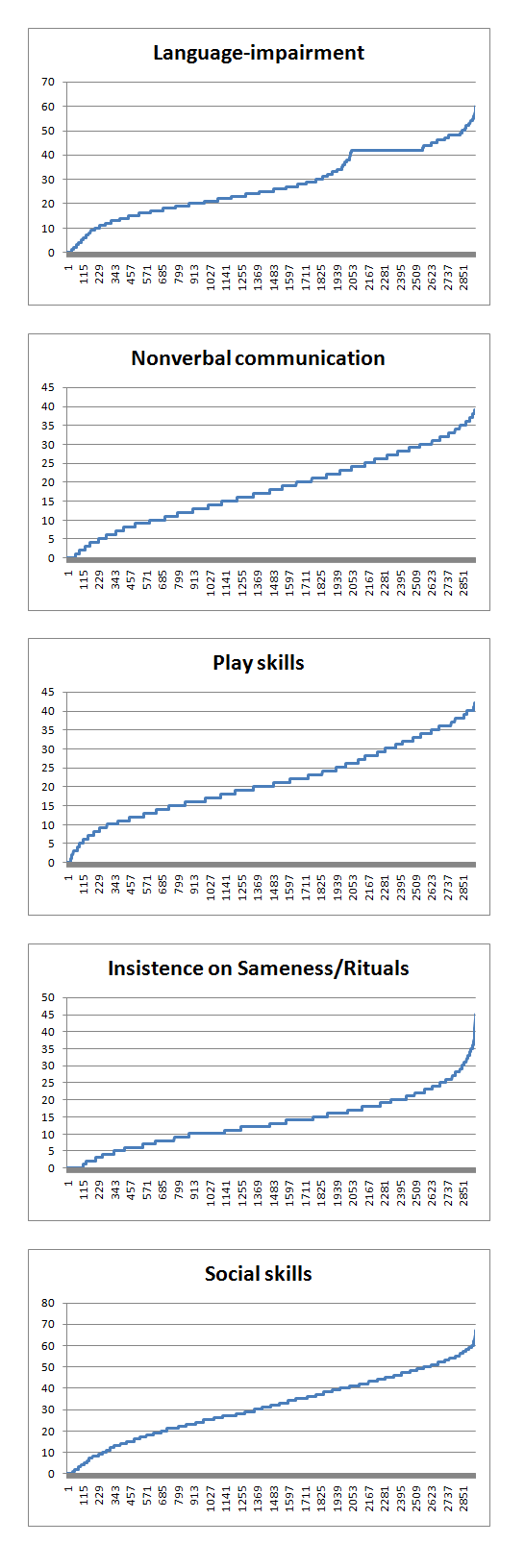

Supplement: Figure S1 — Quantitative trait profiles generated by summing the severity scores for ADI-R items for each trait listed in Table S1. (TIF) [file pone.0019067.s001.tif]

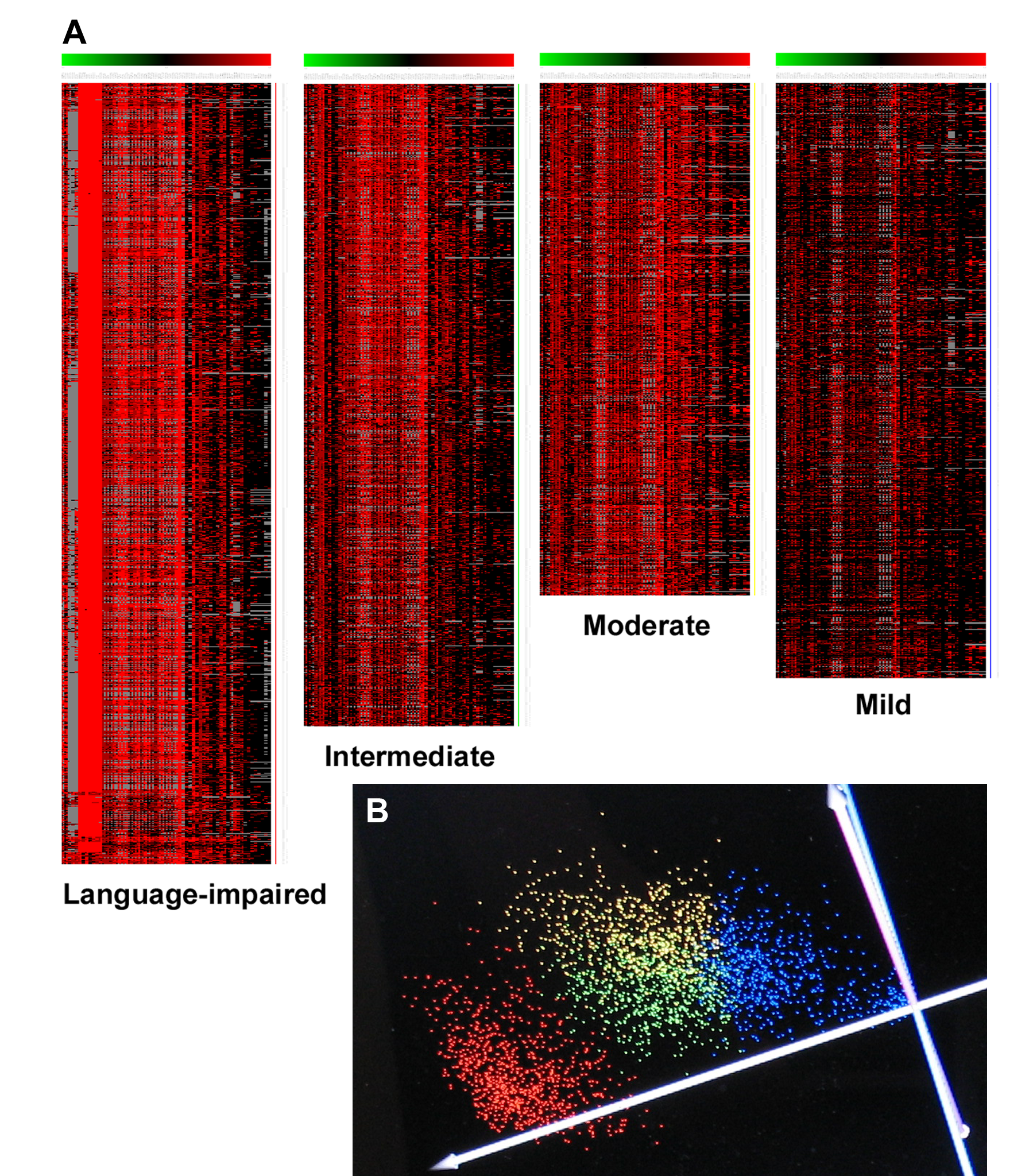

Supplement: Figure S2 — Identification of ASD subtypes by cluster analyses. A) Symptomatic profiles of the 4 ASD subtypes that resulted from K-means cluster analyses of 123 ADI-R severity scores per individual. In this figure, each row represents an individual and each column represents an item on the ADI-R. Black represents a score of 0 which is considered “normal,” while the intensity of red indicates severity scores ranging from 1–3. Gray represents unavailable data. The wide band of intensely red items in the language-impaired subgroup corresponds to spoken language. The 12 columns at the extreme right in each block represent items corresponding to “Savant skills,” which appear to be present at a slightly higher frequency in the group labeled “Moderate.” This group had been labeled “Savant” in our previous study [13]. Note that each cluster contains an independent cohort of cases. B) Principal components analysis (PCA) of the individuals based on the 123 ADI-R severity scores. Each subgroup of individuals identified in (A) is assigned a color, which identifies individuals from that subgroup in the PCA. Red: Language-impaired; Green: Intermediate; Yellow: Moderate; Blue: Mild. Each point on the PCA represents an individual with an ASD whose position is defined by his/her scores for the 123 ADI-R items. (TIF) [file pone.0019067.s002.tif]

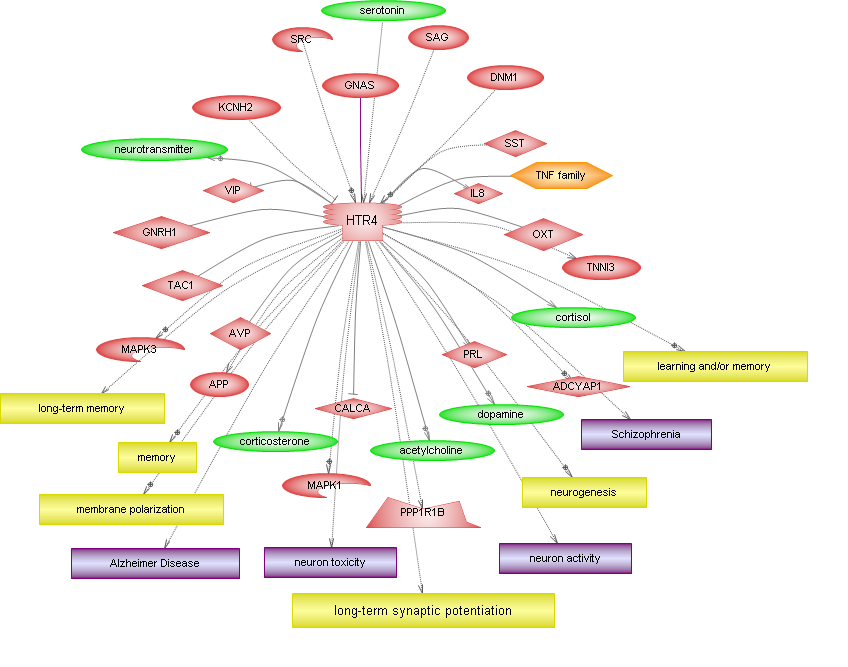

Supplement: Figure S3 — Network connections centered on HTR4 from Figure 3 . (TIF) [file pone.0019067.s003.tif]

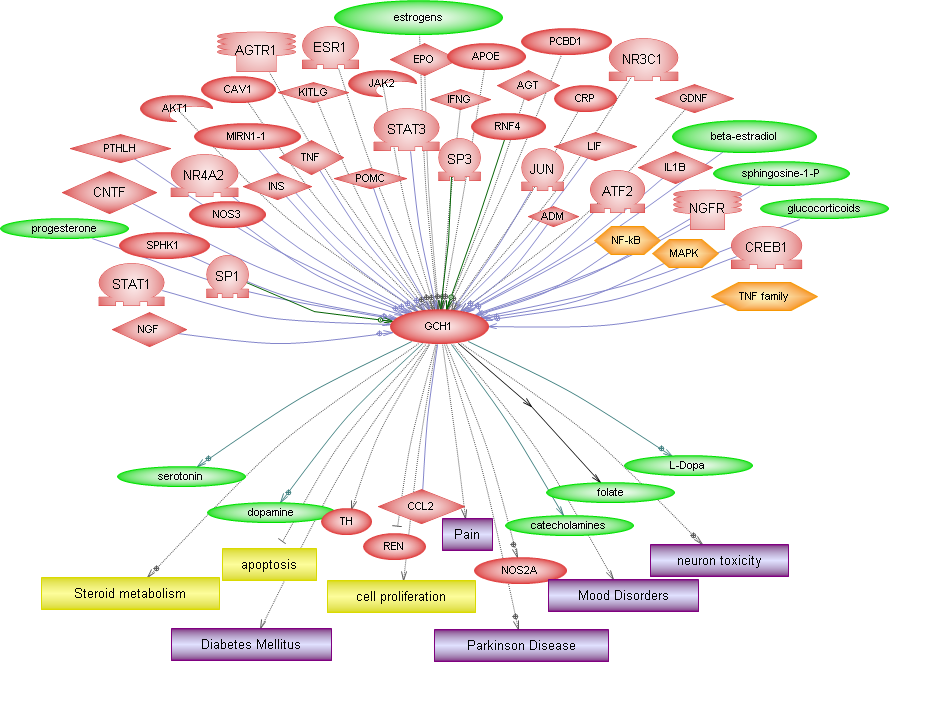

Supplement: Figure S4 — Network connections centered on GCH1 from Figure 3 . (TIF) [file pone.0019067.s004.tif]
