# Supplementary material for: Novel Autism Subtype-Dependent Genetic Variants Are Revealed by Quantitative Trait and Subphenotype Association Analyses of Published GWAS Data
Source: PLoS One. 2011 Apr 27;6(4):e19067. doi: 10.1371/journal.pone.0019067 (PMC3083416; doi:10.1371/journal.pone.0019067)
Supplement: Table S1 — List of behavioral categories and associated ADI-R items used for quantitative trait (QT) analyses. (DOC) [file pone.0019067.s005.doc]

**Table S1.** List of behavioral categories and associated ADI-R items used for quantitative trait (QT) analyses.
